# Kimura’s disease affecting the axillary lymph nodes: a case report

**DOI:** 10.1186/s12893-017-0260-8

**Published:** 2017-05-26

**Authors:** Kenji Kuroda, Shinichiro Kashiwagi, Hitoshi Teraoka, Haruhito Kinoshita, Mikio Nanbara, Eiji Noda, Takaaki Chikugo, Kosei Hirakawa, Masaichi Ohira

**Affiliations:** 1 Department of Surgery, Baba Memorial Hospital, Higashi 4-244 Hamadera Funao-cho, Nishi-ku, Sakai, Osaka 592-8555 Japan; 2 Department of Surgical Oncology, Osaka City University Graduate School of Medicine, 1-4-3 Asahi-machi, Abeno-ku, Osaka Japan; 3 Department of Pathology, Kinki University Faculty of Medicine, Osaka-sayama, Osaka 589-8511 Japan

**Keywords:** Kimura’s disease, Axillary lymph nodes, Eosinophilic granuloma, IgE-RIST, Biopsy

## Abstract

**Background:**

Kimura’s disease (KD; eosinophilic granuloma of soft tissue) is an inflammatory granulomatous disorder of unknown cause with eosinophilic infiltration that occurs mainly in soft tissue. KD occurs mainly in the head and neck, but development in the axillary region is very rare.

**Case presentation:**

A 74-year-old Japanese woman was evaluated for a mass that she noted in the left axillary region. On physical examination, there was a palpable, thumb-sized, hard, elastic, freely movable mass in the left axilla. Blood tests showed elevated soluble interleukin-2 receptor (sIL-2R), normal serum immunoglobulin (Ig) G4, and elevated serum IgE. Ultrasonography of the left axilla showed multiple lymph nodes (LNs) with irregular margins in which central hyperechogenicity was lost. A systemic search by computed tomography (CT) showed no systemic lymphadenopathy or other mass-like lesions suspicious for a primary tumour other than in the left axillary LNs. Biopsy of an excised LN was performed under local anaesthesia for a definitive diagnosis. Histopathology showed various-sized lymphoid follicles, large nodular lesions with an enlarged mantle zone, multiple various-sized germinal centres in single nodules, and eosinophilic infiltration between the nodes. Immunohistochemical (IHC) staining of the germinal centres was positive for cluster of differentiation (CD) 10, positive for B-cell lymphoma (bcl)-6, and negative for bcl-2. These findings led to a diagnosis of KD. Ultrasound after 3 months of follow-up showed disappearance of the axillary lymphadenopathy.

**Conclusions:**

A very rare case of KD in the axillary LNs was described. KD has the potential to occur in any region.

## Background

Kimura’s disease (KD; eosinophilic granuloma of soft tissue) is an inflammatory granulomatous disorder of unknown cause with eosinophilic infiltration that occurs mainly in soft tissue. KD presents clinically as pruritic and pigmented lymph nodes (LNs) or painless masses in soft tissue [1]. KD occurs mainly in the head and neck, but development in the axillary region is very rare [2]. This report describes a patient with KD in the axillary along with a review of the relevant literature.

## Case presentation

A 74-year-old Japanese woman was evaluated for a mass that she noted in the left axillary region. Her past and medication histories were unremarkable. On physical examination, there was a palpable, thumb-sized, hard, elastic, freely movable mass in the left axilla. No breast masses or skin lesions were noted. Blood tests showed a normal white blood cell count, but a differential showed mildly elevated eosinophils (6%). Biochemical tests showed mildly elevated alkaline phosphatase (ALP) of 326 IU/l (normal, 60-200 IU/l), carcinoembryonic antigen (CEA) of 2.3 ng/ml (normal, <5.0 ng/ml), cancer antigen 15-3 (CA15-3) of 10.0 IU/ml (normal, <30.0 IU/ml), National Cancer Center-stomach-439 (NCC-ST-439) of 3.3 U/ml (normal, <7.0 U/ml), and elevated soluble interleukin-2 receptor (sIL-2R) of 604.9 U/ml (normal, 122.0–496.0 U/ml). Serum immunoglobulin (Ig) G4 was normal at 47.7 mg/dl (normal, 4.8–105.0 mg/dl), but immunoglobulin E-radioimmunosorbent test (IgE-RIST) results were elevated at 3032 IU/ml (normal, <173.0 IU/ml).

**Fig. 1:**
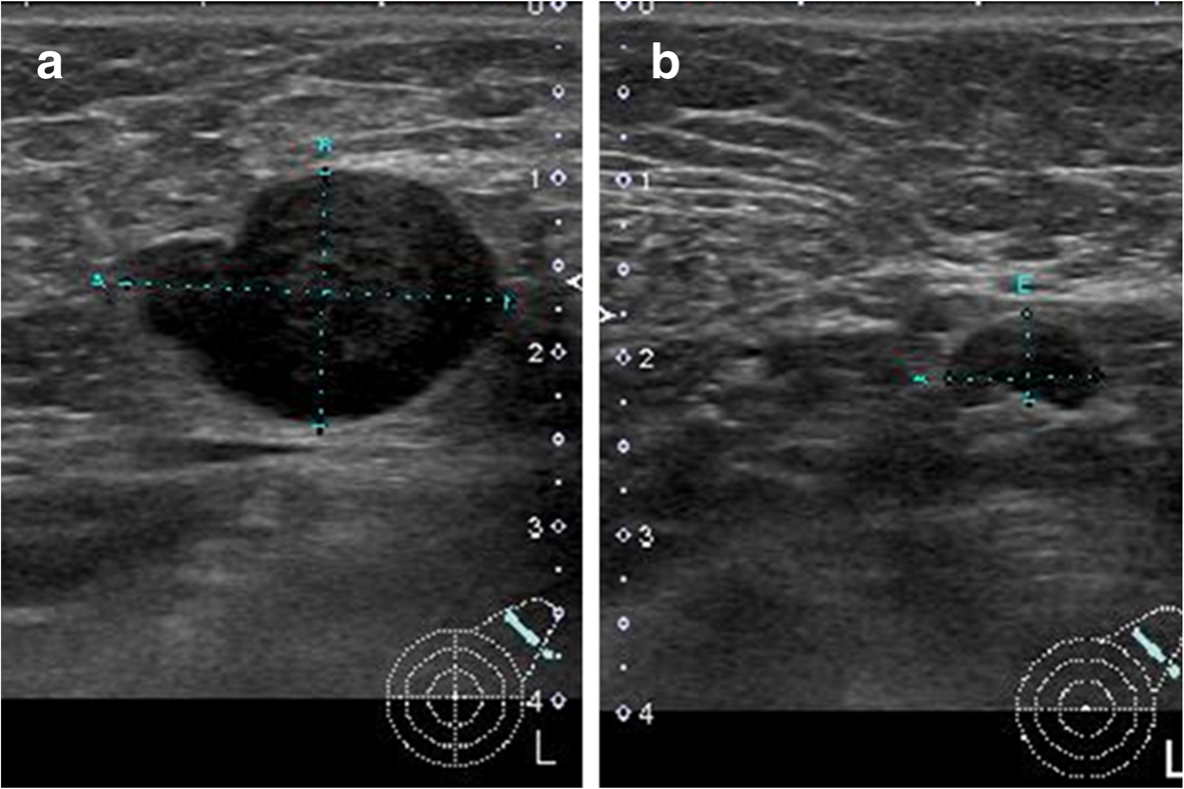
Ultrasound findings: Ultrasound of the *left axilla* showed multiple LNs with *irregular margins* in which central hyperechogenicity was lost (maximum size: 2.2 cm × 1.5 cm; **a**, **b**)

**Fig. 2:**
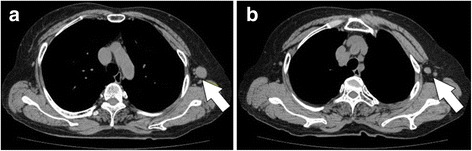
CT image findings: A systemic search using computed tomography (CT) showed multiple lymphadenopathy only in the *left axilla*, with no other systemic lymphadenopathy or mass-like lesions suspicious of a primary tumour (**a**, **b**)

**Fig. 3:**
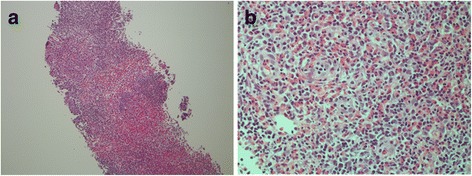
Pathological findings of core needle biopsy: Pathological examination of the biopsy specimens showed LNs with rich eosinophilic infiltration, but no evidence of any atypical cells (**a**, ×40; **b**, ×400)

Ultrasonography (US) of the left axilla showed multiple LNs with irregular margins in which central hyperechogenicity was lost (maximum size: 2.2 cm × 1.5 cm) (Fig. 1a, b). A systemic search using computed tomography (CT) showed multiple lymphadenopathy only in the left axilla, with no other systemic lymphadenopathy or mass-like lesions suspicious of a primary tumour (Fig. 2a, b). To differentiate a malignancy such as malignant lymphoma or LN metastases from an unknown primary tumour, a core needle biopsy (CNB) of the left axillary LNs was performed. Pathology examination of the CNB showed LNs with rich eosinophilic infiltration but no evidence of any atypical cells (Fig. 3a, b).

**Fig. 4:**
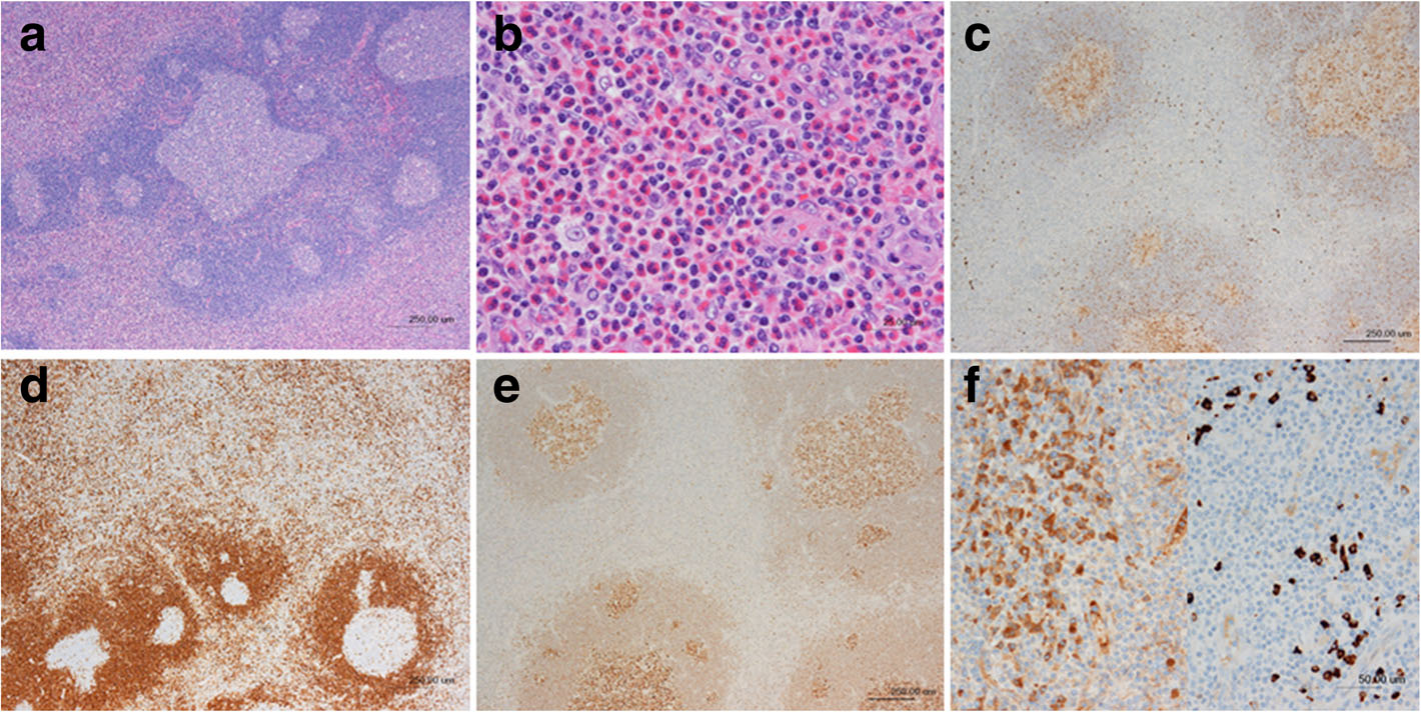
Pathological findings of the excised lymph nodes: Histopathology showed various-sized lymphoid follicles and large nodular lesions with an enlarged mantle zone, and multiple germinal centres in single nodules (**a**). Proliferation of small blood vessels and marked eosinophilic infiltration were also observed between the nodes (**b**). Immunohistochemical staining of the germinal centres was positive for CD10 (**c**), negative for bcl-2 (**d**), and positive for bcl-6 (**e**). Staining was positive for IgG in the germinal centres and between nodes, and some areas were IgG4-positive (**f**)

Immunohistochemical (IHC) staining showed no evidence of malignant lymphoma or LN metastases from an occult breast cancer (cytokeratin (CK) 7-negative, CK20-negative, and cluster of differentiation (CD) 117-negative). Biopsy of an excised LN was performed under local anaesthesia for a definitive diagnosis. Histopathology showed various-sized lymphoid follicles, large nodular lesions with an enlarged mantle zone, and multiple germinal centres in single nodules (Fig. 4a). Proliferation of small blood vessels and marked eosinophilic infiltration were also observed between the nodes (Fig. 4b). IHC staining of the germinal centres was positive for CD10, negative for B-cell lymphoma (bcl)-2, and positive for bcl-6 (Fig. 4c–e). Staining was positive for IgG in the germinal centres and between nodes, and some areas were IgG4-positive (Fig. 4f).

An IgG4-related disease was also considered, but serum IgG4 levels were normal. The findings eventually led to a diagnosis of KD. The patient was followed up, in part based on her own wishes, and an ultrasound performed 3 months later showed disappearance of the axillary lymphadenopathy. As of this writing, 6 months after the initial examination, there has been no recurrence of KD.

## Discussion

KD is a chronic inflammatory granulomatous disorder with the formation of masses characterized by eosinophilic infiltration and lymphoid follicular hyperplasia in subcutaneous soft tissue. KD was first reported by Kimura et al. in 1948 [3]. KD has a predilection for Asian males in their 30s and 40s and presents clinically as single or multiple masses in subcutaneous soft tissue and LNs; although the masses are often painless, they may be associated with pruritus and skin pigmentation [2]. These masses mostly occur in the head and neck, and although the limbs and inguinal regions are sometimes affected, occurrence in the axillary region is rare [2, 4, 5]. Additionally, there are case reports that KD involving the thoracic and abdominal lymph nodes may be confused with malignant lymphoma or other metastatic cancers [6, 7]. Therefore, KD has the potential to occur in any region.

The blood tests used to diagnose KD typically show peripheral blood eosinophilia and elevated serum IgE levels [1]. Histopathology shows various-sized germinal centres with lymphoid follicular hyperplasia, surrounding eosinophilic infiltrates, and blood vessels with vascular endothelial cell swelling. IHC staining is often positive for IgE in the germinal centres of follicles [8–10].

The present patient was an elderly woman who initially presented with enlarged axillary LNs. However, considering the rarity of KD and its epidemiologic features, it was not initially suspected. The differential diagnosis of lymphadenopathy included LN metastases due to occult breast cancer, malignant lymphoma, reactive lymphadenitis, IgG4-related disease, and eosinophilic granuloma. Therefore, a detailed pathological examination was performed to make a definitive diagnosis. Haematoxylin-eosin (HE) staining showed lymphoid follicular hyperplasia suggesting progressively transformed germinal centres and germinal centres of varying sizes with eosinophilic infiltration between the follicles. The morphologic features suggested KD or IgG4-related disease, but the blood test results showing mild peripheral blood eosinophilia, elevated serum IgE, and normal serum IgG4 ultimately led to a diagnosis of KD.

No definitive therapy for KD has been established, but the use of surgical excision, radiation therapy, or drug treatment alone or in combination has been reported. Surgery is usually performed if the masses can be excised, but other treatment may be selected in patients with possible neurovascular injury or masses that are not localized [11]. Radiation therapy is also associated with a relatively low rate of disease recurrence, but adverse reactions may occur, and it might not be indicated for younger patients.

Drug therapy includes steroids, non-steroidal anti-inflammatory and analgesic drugs, and suplatast tosilate (Th2 cytokine inhibitor)*,* but disease recurrence rates are high, and the risk of adverse reactions with long-term use must be considered [11]. The present patient underwent LN excision for a definitive diagnosis, and after a follow-up period, the axillary lymphadenopathy disappeared. However, because KD has a high recurrence rate, long-term follow-up is required.

## Conclusions

A very rare case of KD in the axillary lymph nodes was described. KD has the potential to occur in any region.

## Abbreviations

ALP: Alkaline phosphatase
bcl: B-cell lymphoma
CA15-3: Cancer antigen 15-3
CD: Cluster of differentiation
CEA: Carcinoembryonic antigen
CK: Cytokeratin
CNB: Core needle biopsy
CT: Computed tomography
Ig: Immunoglobulin
IgE-RIST: Immunoglobulin E-radioimmunosorbent test
IHC: Immunohistochemical
KD: Kimura’s disease
LN: Lymph node
NCC-ST-439: National Cancer Center-stomach-439
sIL-2R: soluble interleukin-2 receptor
US: ultrasonography
